# Cause analysis of PM_2.5_ pollution during the COVID-19 lockdown in Nanning, China

**DOI:** 10.1038/s41598-021-90617-5

**Published:** 2021-05-27

**Authors:** Zhaoyu Mo, Jiongli Huang, Zhiming Chen, Bin Zhou, Kaixian Zhu, Huilin Liu, Yijun Mu, Dabiao Zhang, Shanshan Wang

**Affiliations:** 1 Shanghai Key Laboratory of Atmospheric Particle Pollution and Prevention, Department of Environmental Science and Engineering, Fudan University, No. 220 Handan Road, Shanghai, 200433 China; 2 Atmospheric Environment Research Center, Scientific Research Academy of Guangxi Environmental Protection, Nanning, 530021 China; 3 Department of Occupational Health and Environmental Health, School of Public Health, Guangxi Medical University, Nanning, 530021 China

**Keywords:** Environmental sciences, Natural hazards, Planetary science

## Abstract

To analyse the cause of the atmospheric PM_2.5_ pollution that occurred during the COVID-19 lockdown in Nanning, Guangxi, China, a single particulate aerosol mass spectrometer, aethalometer, and particulate Lidar coupled with monitoring near-surface gaseous pollutants, meteorological conditions, remote fire spot sensing by satellite and backward trajectory models were utilized during 18–24 February 2020. Three haze stages were identified: the pre-pollution period (PPP), pollution accumulation period (PAP) and pollution dissipation period (PDP). The dominant source of PM_2.5_ in the PPP was biomass burning (BB) (40.4%), followed by secondary inorganic sources (28.1%) and motor vehicle exhaust (11.7%). The PAP was characterized by a large abundance of secondary inorganic sources, which contributed 56.1% of the total PM_2.5_ concentration, followed by BB (17.4%). The absorption Ångström exponent (2.2) in the PPP was higher than that in the other two periods. Analysis of fire spots monitored by remote satellite sensing indicated that open BB in regions around Nanning City could be one of the main factors. A planetary boundary layer-relative humidity-secondary particle matter-particulate matter positive feedback mechanism was employed to elucidate the atmospheric processes in this study. This study highlights the importance of understanding the role of BB, secondary inorganic sources and meteorology in air pollution formation and calls for policies for emission control strategies.

## Introduction

Air pollution, particularly fine particulate matter (PM_2.5_), has caused significant economic loss and adverse public health effects in countries such as China^1–3^. Regarding severe air pollution issues and to protect public health, the State Council of China promulgated the most stringent Air Pollution Prevention and Control Action Plan (Action Plan) in 2013^4^, in which PM_2.5_ concentration reductions of 25%, 20%, and 15% in 2017 compared with levels in 2013 were mandated in the Beijing–Tianjin–Hebei (BTH), Yangtze River Delta (YRD) and Pearl River Delta (PRD) areas, respectively. Tremendous efforts include setting more restricted industrial and vehicle emission standards, closing down heavy pollution factories, and upgrading industrial facilities to mitigate various pollutant emissions. Evaluation of the effectiveness of these measures can provide crucial information for developing clean-air strategies in China as well as in other developing countries facing similarly severe pollution issues^5^.

Due to the coronavirus disease 2019 (COVID‐19) pandemic occurring at the end of 2019^6^, the epidemic centre of Wuhan city announced a lockdown on January 23, 2020. China implemented a national emergency response to control the spread of the disease^7^, resulting in a significant reduction of primary emissions, such as from industrial operations, motor vehicle exhaust, construction operations and smoke from restaurants^8,9^. The COVID-19 lockdown creates a unique and valuable opportunity to assess human activities on local air quality and to understand the factors affecting air pollutants. Many studies^8–17^ have reported the environmental effects of lockdown policies in different regions due to the COVID‐19 pandemic. The effect of air quality in different stages of the COVID‐19 pandemic and in different regions varied. In general, more consistent NO_2_ declines compared with other pollutants coincided with reduced vehicular emissions according to previous studies^8–17^. CO^10,17^ and black carbon (BC)^14,18^ also significantly declined following the COVID-19 lockdown period because of vehicle emission reduction. However, the observed reductions in PM_2.5_ were found to be significantly less than those in NO_*2*_ in most cities studied^14–16^. Even in some regions, PM_2.5_ increased or pollution events have been reported^8,9,16^. Li et al.^8^ indicated that even during the lockdown, with a primary emission reduction of 15–61%, the daily average PM_2.5_ concentrations in the YRD still ranged between 15 and 79 μg/m^3^, which showed that background and residual pollutants were still high.

There are still large uncertainties in quantifying the sources of PM_2.5_ at the citywide scale due to the synergetic effects of complex aerosol chemistry and transboundary transport^12^. The driving factors of PM_2.5_ explosive growth events include the secondary aerosol formation of sulphate and nitrate and primary emissions, but this secondary transformation contributes more to explosive growth events^19^. Several studies^20–23^ have shown that severe air pollution events have been successfully mitigated by controlling anthropogenic emissions in China. For instance, a statistical model was developed and indicated that the implementation of stringent emission reduction measures alone could effectively lower PM_2.5_ levels by 20–24 μg/m^3^ (27–33%) on average during the 2008 Beijing Olympic Games^20^. In addition, some other studies have also shown that meteorological factors play critical roles in the formation of atmospheric pollution incidents and should be taken into account to determine the actual influence of controlling procedures on pollution reduction^9,23–25^^.^

Surprisingly, from 18 to 24 February 2020, a regional PM_2.5_ pollution incident took place in Guangxi, southern China, and slight atmospheric pollution was observed in the provincial capital of Nanning City on 20 February. During the COVID-19 lockdown period, regular air pollutant emissions from industrial and domestic activities decreased significantly due to reduced road motor vehicle volumes and postponed infrastructure construction^8,25,26^. This pollution incident was unexpected and caused social concern. Similarly, despite the extreme reductions in primary emissions in Guangxi during the COVID-19 lockdown, the current air pollution could not be fully addressed. Thus, it is important to study the precise factors that caused the atmospheric pollution formation.

How to control air pollution remains a challenge because of the complexities of pollutant sources, atmospheric chemistry, and meteorology^17^. Hence, we aim to assess the contribution of pollutant sources to PM_2.5_ and the influencing factors of related atmospheric physiochemistry and meteorological conditions in this study. The atmospheric pollution incident in Nanning was selected as a case investigation for pollution source analysis utilizing multiset equipment installed in an atmospheric observatory station. A single particulate aerosol mass spectrometer, aethalometer, particulate Lidar, surface meteorological and environmental data, satellite remote sensing data and modelled HYSPLIT4 trajectory were used to analyse the cause of the PM_2.5_ pollution in Nanning during the COVID-19 control period, when a regional haze pollution episode occurred from February 18 to February 24 in Guangxi. The results in this study provide a reference for future air pollution control in cities in southern China.

## Results and discussion

### Overall regional pollution situation in Guangxi

From 18 to 24 February 2020, a regional air pollution event with a cumulative pollution period of 1.1 days occurred in Guangxi, and the key pollutant was PM_2.5_ (see Supplementary Fig. S1). During this period, this pollution event first started on 19 February, and slight pollution [the air quality index (AQI) > 100, PM_2.5_ > 75 μg/m^3^] was identified in four cities; namely, Laibin, Chongzuo, Liuzhou and Hechi; among them, Chongzuo presented the highest AQI value (AQI = 123). However, the most severe pollution was observed in Hechi City on 20 February (AQI > 200, PM_2.5_ > 150 μg/m^3^); then, the levels of pollutants began to decline (with a slight increase on 23 February) and the event finally ended on February 24.

To evaluate the regional overall pollution status in Guangxi during this period, a comparison of average concentrations of atmospheric PM_2.5_ in Guangxi during 19–23 February 2020 with those in all other provinces is illustrated in Supplementary Fig. S2. The average PM_2.5_ concentration was 61 μg/m^3^, which was the third highest nationwide on these days (the average PM_2.5_ concentrations in Hechi City even reached 79 μg/m^3^). In contrast, the average atmospheric PM_2.5_ concentrations in the surrounding provinces, e.g., Guangdong, Yunnan, Guizhou and Hunan, were only 31, 35, 44 and 46 μg/m^3^, respectively, which were significantly lower than that in Guangxi. This indicated that the pollutants may have mainly originated from local sources. Interestingly, although the influence of industrial and domestic activities had been reduced to a minimum extent within Guangxi or in neighbouring provinces during the COVID-19 lockdown, the regional pollution event in Guangxi did take place. Accordingly, we conducted a cause analysis of this PM_2.5_ pollution event based on data from different types of equipment installed at the Scientific Research Academy of Guangxi Environmental Protection (SRAGEP).

### Data representativeness at the sampling site

To evaluate the representativeness of the data measured at the sampling site, the homochronous data released by the national real-time municipal air quality platform (http://106.37.208.233:20035) of the China National Environmental Monitoring Centre (CNEMC) were employed for comparison (see Supplementary Table S1). The daily average values of PM_2.5_, PM_10_, SO_2_, NO_2_, O_3_ and CO were relatively consistent within these two monitoring systems. The correlation analysis of hourly PM_2.5_ and CO data is shown in Supplementary Fig. S3. The correlation coefficients for the two pollutants were both above 0.93, which indicated an excellent representativeness of data recorded at the station for the ambient air quality at the SRAGEP in reference to CNEMC.

### Overall air pollution situation in Nanning City

**Figure 1:**
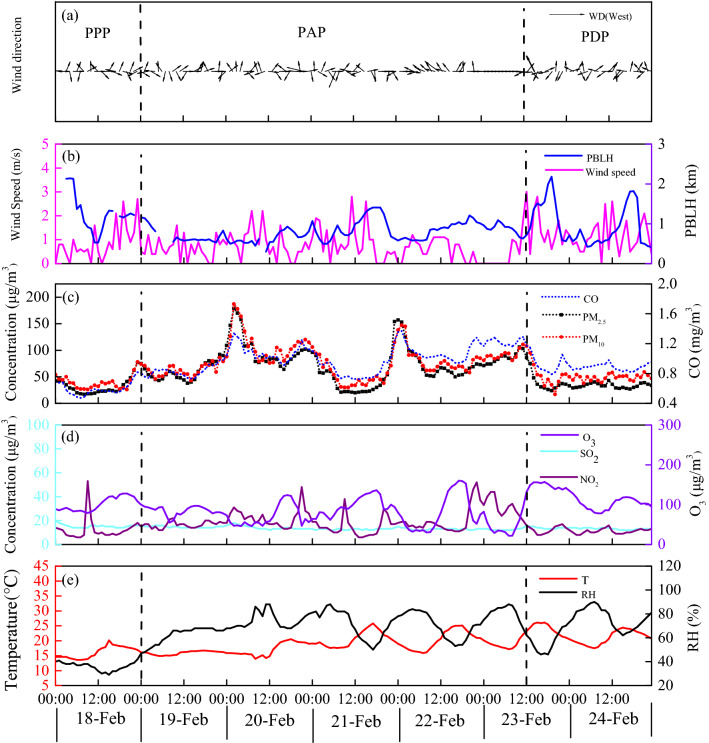
Time series of (**a**) wind direction (WD); (**b**) wind speed (WS) and planetary boundary layer heights (PBLH); (**c**) the main pollutants (PM_10_, PM_2.5_ and CO); (**d**) the main gaseous pollutants (O_3_, SO_2_, and NO_2_); and (**e**) meteorological conditions (temperature and RH) during the study period at the SRAGEP site.

The time series of mass concentrations of atmospheric pollutants along with key meteorological conditions in Nanning city from 18 to 24 February 2020 are illustrated in Fig. 1. According to the PM_2.5_ concentration variations, the total progression of pollution could be divided into three stages, i.e., a pre-pollution period (PPP, February 18), pollution accumulation period (PAP, February 19 to 12:00 on February 23) and pollution dissipation period (PDP, 12:00 on February 23 to February 24). The corresponding average PM_2.5_ concentrations for the PPP, PAP and PDP were 32, 73, and 35 μg/m^3^, respectively. The PM_2.5_ concentration during the PAP was twice as high as that during the other two stages. Similarly, the average concentrations of CO in the PAP (1.00 mg/m^3^) were also higher than those in the PPP and PDP (0.57 and 0.90 mg/m^3^, respectively). Interestingly, while the PAP and PDP possessed average relative humidities (RHs) of 71% and 70%, respectively, the average RH in the PPP was only 36%. The main reason for this could have been the lower wind speed (WS) in the PAP (0.71 m/s) than in the PPP (0.87 m/s) and PDP (1.19 m/s). Additionally, the average planetary boundary layer heights (PBLHs) were 1253 m, 787 m and 987 m in the PPP, PAP and PDP, respectively. Moderate atmospheric pollution was identified in Nanning city on 20 February with a PBLH ranging from 297 to 934 m on that day. Generally, PBLH is higher during the daytime, especially at noon; however, a minimum value of the PBLH (297 m) was observed at 11 a.m. on 20 February, which indicates that the potential adverse meteorological conditions for the horizontal and vertical diffusion of pollutants could have resulted in the high PM_2.5_ concentration on that day. However, during the PDP, particularly on 24 February, the atmospheric diffusion conditions were in favour due to the low air pressure near the ground and airflow currents towards the southerly direction; thus, the calm weather remaining for several days was mitigated through the enhanced airflow conditions, and as a result, the air quality began to improve.

### Single particle source analysis

**Figure 2:**
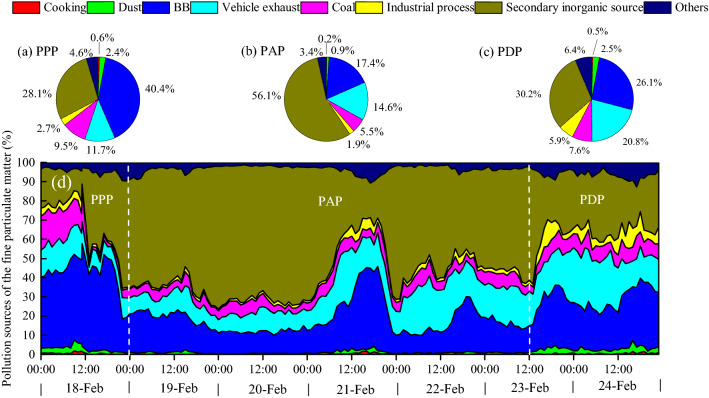
Temporal change for the percentage of single particle source resolution.

By analysing the composition of particulate matter, the sources of PM_2.5_ in municipal areas could be categorized into cooking, dust, biomass burning (BB), vehicle exhaust, coal, industrial processes (noncombustion processes), secondary inorganic sources and others (Fig. 2). The dominant source of PM_2.5_ in the PPP stage was BB (accounting for 40.4%), followed by secondary inorganic sources (accounting for 28.1%) and motor vehicle exhaust (11.7%). In contrast, secondary inorganic sources dominated in the PAP (56.1%) and PDP (30.2%), and it seemed that there was an obvious increase in secondary transformed particulates during the PAP. The second largest contribution source of PM_2.5_ in the PAP and PDP was BB, which accounted for 17.4% and 26.1%, respectively. By analysing the fire spot map from satellite remote sensing monitoring, large numbers of fire spots were identified in Nanning city and the surrounding regions; however, no significant pollution was generated, which was attributed to relatively better meteorological diffusion conditions on February 18–19. As the atmospheric horizontal and vertical diffusion conditions worsened afterwards, pollutants began to accumulate and produce more particulates from secondary transformations, which induced more severe PM_2.5_ pollution on February 20.

The dominant species of secondary inorganic aerosols (SIAs) were sulphate and nitrate. Heterogeneous reactions and gas-phase oxidation on particle surfaces are the dominant forms of SIAs^19,27^. Under stagnant weather, the rapid conversion of primary gaseous pollutants to secondary aerosols greatly contributes to PM_2.5_ explosive growth^28,29^. Under high relative humidity, low wind speed and a decreased PBLH during the PAP, the formation of sulphate and nitrate by gas-phase oxidation and heterogeneous reactions may have been enhanced. The reason for the high contribution from secondary inorganic sources rather than from BB in the PAP was related to the accumulation of air pollutants and the enhanced secondary transformations under stagnant conditions^19,30^.

**Figure 3:**
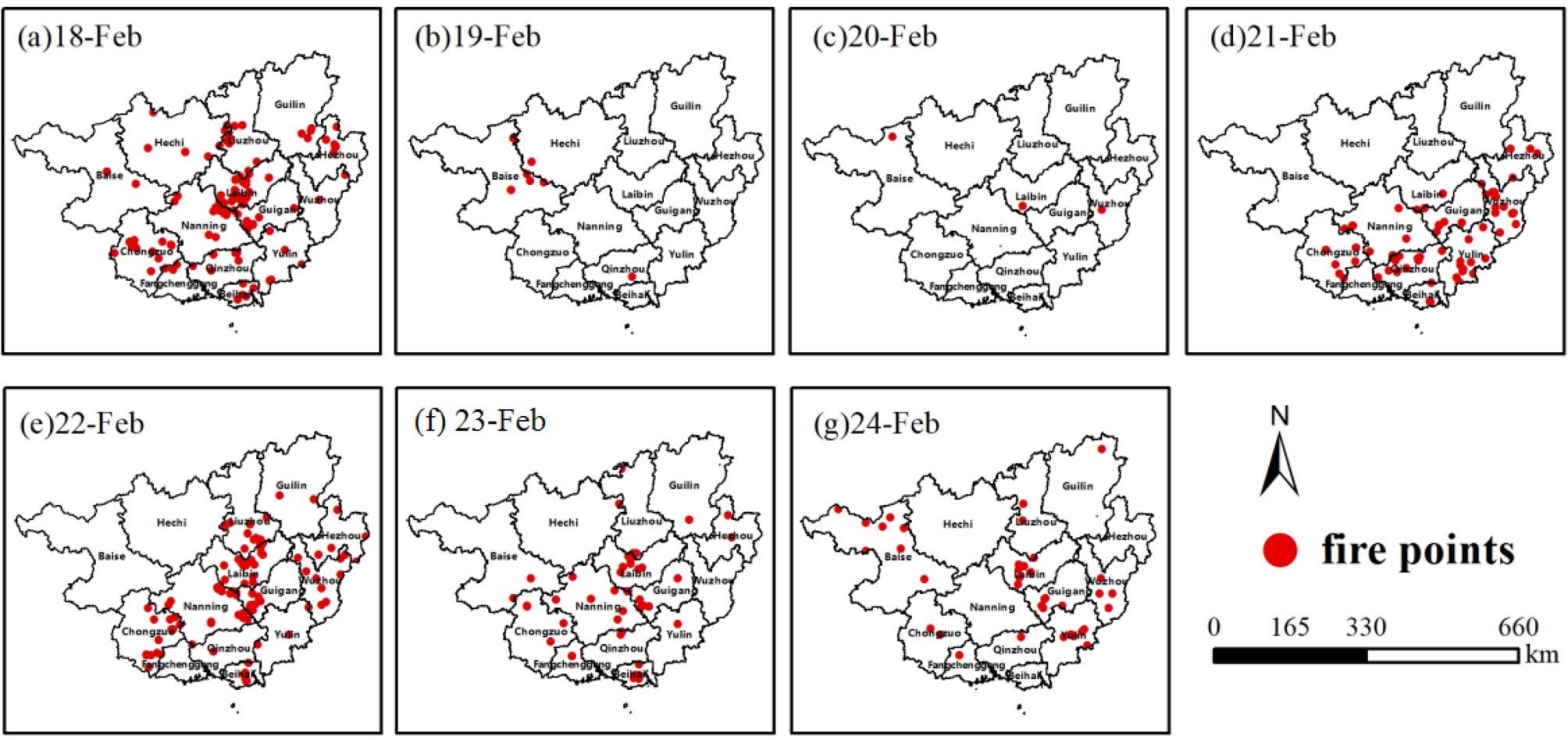
Distribution of fire spots in Guangxi from February 18 to 24, 2020. The map was generated using ArcGIS Desktop 10.3, https://desktop.arcgis.com/en. (Data source of fire spots: Satellite-monitored fire spots (Aqua, Himawari-8, NOAA20, NPP and Terra).

As discussed above, BB was confirmed to be the largest contributor of pollution during the PPP and the second largest contributor in the other two stages. Fire spot maps were constructed based on satellite remote sensing data (Fig. 3), and straw incineration fire spots were identified from February 18 to 24. The total count of straw fire spots in Guangxi was 421, and 52 of them were in Nanning City, while 70, 45 and 44 fire spots were distributed in three cities bordering Nanning (Laibin, Chongzuo and Guigang, respectively). The fire spots in these four cities were the most intensified among the 14 cities in Guangxi during the observation period, which implied that the pollution in Nanning was attributed to the regional BB. As the three largest sugar producing cities in Guangxi, Nanning (No. 1), Laibin (No. 2) and Chongzuo (No. 3) possess large areas of sugarcane farming that produce large amounts of waste sugarcane leaves. The open-air incineration of sugarcane leaves induces significant difficulties in regional air pollution reduction, particularly urban atmospheric pollution control. It has been reported that the incineration of agricultural straw causes significant effects on air quality, public health and climate in China^31–33^. In northern China, pollution due to straw incineration usually takes place in autumn^34^, for instance, open BB contributed 52.7% of atmospheric PM_2.5_ in the northeastern region of China during November 1–4, 2015. However, there is little research on how BB affects air quality in southern China. Guangxi has the highest pollutant emissions from BB among the 31 provinces in China^35^. During the COVID-19 epidemic control period, pollution from other sources, such as industry and automobile vehicles, significantly decreased^8,36,37^. Although harvesting of sugarcane in 2019/2020 occurred during the COVID-19 lockdown, the activities of straw incineration in vast rural regions continued according to the traditional farming season. The planting area and production of sugarcane in Guangxi account for more than 60% of the entire country^38^. The production of sugar was 6 million tons during the harvesting operation of sugarcane in 2019/2020. The sugarcane harvesting operation spanned from November to March of the following year^39^. During this period, farmers harvested sugarcane to plant sugarcane in the next season. The NO_2_ concentration increased at approximately 12:00 and reached a peak at approximately 18:00, which was consistent with the practice of farmers predominantly burning straw in the afternoon.

### Statistics of the aerosol light absorption coefficient and AAE

Mean values of aerosol light absorption coefficients σ_abs_ at seven wavelengths and the AAE during different stages are summarized in Table 1. During the COVID-19 strict control period, the light absorptions of both ultraviolet (σ_abs,370_) and infrared (σ_abs,880_) wavelengths in Nanning were significantly lower than data reported in Xiamen^40^ and Lhasa^41^.

**Table 1:** Aerosol light absorption properties.

Stages	σ_abs,370_ (Mm^-1^)	σ_abs,470_ (Mm^-1^)	σ_abs,520_ (Mm^-1^)	σ_abs,590_ (Mm^-1^)	σ_abs,660_ (Mm^-1^)	σ_abs,880_ (Mm^-1^)	σ_abs,950_ (Mm^-1^)	AAE
PPP	23.5	12.5	9.6	7.6	6.3	3.5	3.0	2.2
PAP	15.3	8.9	7.0	5.7	4.7	2.7	2.3	2.0
PDP	3.8	2.5	2.0	1.7	1.4	0.8	0.7	1.7

It should be noted that the value of the AAE could be used as an indicator of aerosols from burning processes of fossil fuel or biomass^42^, as the major content of fossil fuel burning is black carbon (BC); thus, the AAE of aerosols caused by fossil fuel burning is close to 1^43,44^. In contrast, aerosols from BB contain rich amounts of brown carbon (BrC); therefore, they could generate even larger AAEs than those from fossil fuel^42^. In this case, the AAE value of aerosols from the BB was usually larger than 2.0^45^. In this study, the AAEs during the PPP and PAP were 2.2 and 2.0, respectively, which implied that aerosol absorption in Nanning city could be influenced by the burning of both biomass and fossil fuel. Additionally, considering the significant reduction in fossil fuel consumption during the special control period of the new corona epidemic, BB could have been the dominant factor resulting in the higher AAE in the PPP and PAP, which was also supported by the large number of fire spots detected by satellites (commercial data) (Fig. 3).

### Impact analysis of secondary pollution

**Figure 4:**
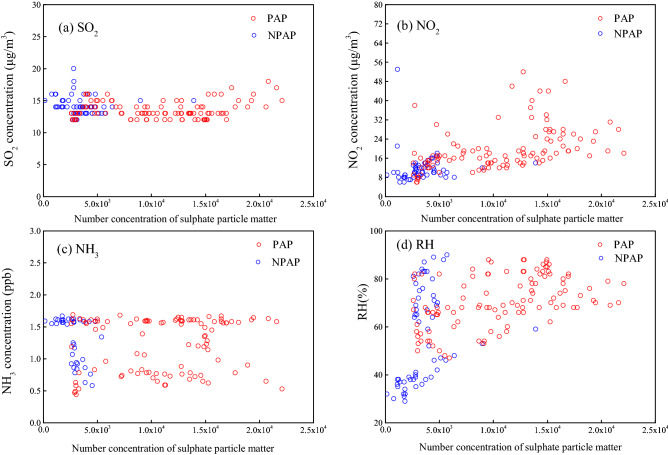
Number concentration of sulphate particulates versus (**a**) concentration of SO_2_; (**b**) concentration of NO_2_; (**c**) concentration of NH_3_ and (**d**) RH during the pollution accumulation period (PAP) and non-pollution accumulation period (NPAP, i.e. pre-pollution and pollution dissipation period) from February 18 to 24, 2020.

The formation of secondary particulate matter may have also significantly contributed to this pollution event, and this conclusion was based on the following two reasons. The first is the synergistic effects of NO_2_ and NH_3_ during high RH on promoting the liquid-phase oxidation of SO_2_, which would have led to an obvious increase in sulphate particulates during the PAP. Figure 4 shows the relationships between sulphate particle number concentration and the levels of SO_2_, NO_2_, NH_3_ and RH, and it seems that positive correlations between the concentrations of sulphate particulates and NO_2_, NH_3_, and RH were identified during the PAP with relatively good linearity. However, before and after the PAP (PPP and PDP), there was no significant positive linear correlation even between SO_2_ and sulphate particulates (Table S2). During the entire observation period, the concentration of SO_2_ remained at a relatively stable level, possibly due to the reduction in industrial emissions and the absence of obvious SO_2_ sources around the observation site in the central district of Nanning city, and there was no significant increase in the SO_2_ concentration even during the PAP when the height of the boundary layer decreased. Interestingly, the concentrations of sulphate particulates showed positive correlation relationships with SO_2_ (r = 0.240), NO_2_ (r = 0.454), NH_3_ (r = 0.116) and RH (r = 0.474) during the PAP. A recent study has proposed that the coexistence of NO_2_ and NH_3_ could promote the conversion of SO_2_ to sulphate particulates under high RH conditions^46^. Additionally, Wang et al.^47^ adopted density function theory (DFT) to investigate how H_2_O and NH_3_ promoted the oxidation reaction of SO_2_ and NO_2_ and found that NH_3_ played an important role in stabilizing the complex products. Chen et al.^48^ employed glassware reactors and demonstrated that NH_3_ could increase the dissolution of SO_2_ and hence enhance the liquid-phase oxidation of SO_2_.

Second, the increase in secondary particulates could be explained by the positive feedback mechanism of pollution boundary layer (PBL)–relative humidity (RH)–secondary particle matter (SPM)–particulate matter (PM), which has been proposed in previous studies, and the relative humidity (RH) and PBLH are essential factors that may affect the formation of atmospheric PM_2.5_^49,50^. The positive feedback mechanism of PBL–RH–SPM–PM suggests that PM levels and RH could be low while the PBLH is high at the beginning stage of a haze event. Since atmospheric diffusion conditions can be adversely impacted by weather conditions, PM begins to accumulate, and radiation effects due to the increase in PM can cause the PBLH to decrease and further induce increases in PM and RH. On the other hand, the moisture absorbed by PM would increase and enhance the radiation effect, which could further decrease the PBLH. Thus, RH would keep increasing and accordingly enhance the formation of SPM. Therefore, through the comprehensive analysis of the extinction coefficient, PBLH, PM and RH, which were observed by LiDAR, it is concluded that the PBL–RH–SPM–PM positive feedback mechanism is an ideal model to elucidate the occurrence and development of pollution.

**Figure 5:**
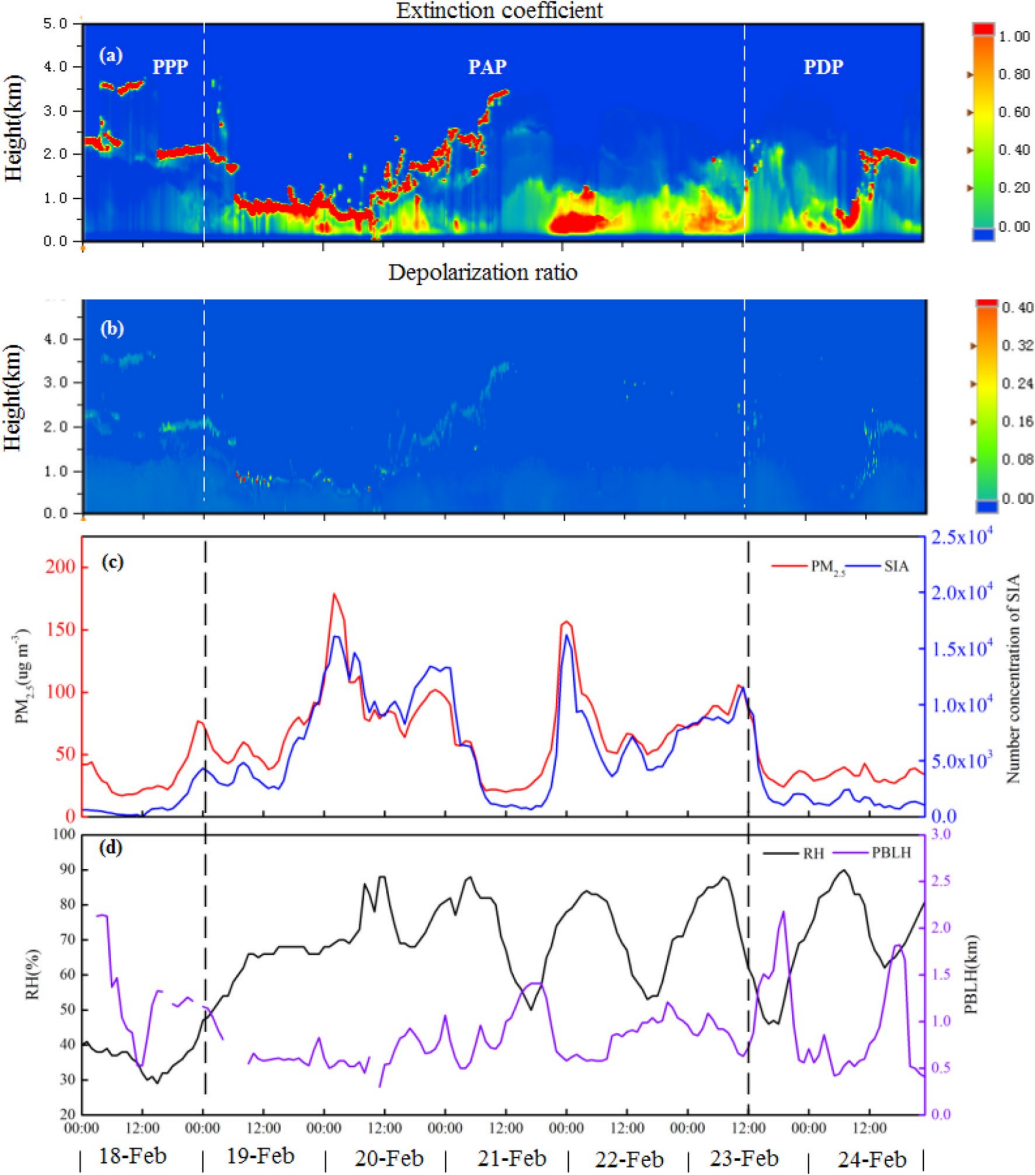
(**a**,**b**) The time series of the vertical distribution of the extinction coefficient and depolarization ratio of the lidar at 532 nm from February 18 to 24, 2020, respectively, and (**c**) is the time sequence diagram of PM_2.5_ mass concentration and secondary inorganic particle number concentration in the same period. (**d**) is a diagram of RH and PBLH from February 18 to 24, 2020.

Figure 5a,b shows the time series of the vertical distribution of the extinction coefficient and depolarization ratio of the lidar at 532 nm from February 18 to 24, 2020, respectively. This figure demonstrates that the near-ground extinction coefficients for Nanning were relatively small in the PPP and gradually increased. The depolarization ratio was relatively small with little variation for the entire observation period. The PBLH gradually decreased from approximately 2 to 1 km, which was adverse for atmospheric convection and resulted in worse atmospheric diffraction conditions; thus, the pollutants began to accumulate as the concentrations of PM_2.5_ and SIAs levels slowly increased (Fig. 5c,d). Aerosol concentrations are negatively correlated with PBLH^51,52^. Specifically, at 12:00 on February 20 in the PAP, the wind speed was low and the PBLH was relatively low (minimum value was 293 m only), and the atmospheric diffusion condition became even worse and accordingly caused the cumulative accumulation of pollutants. At approximately 0:00 on 20 February, the near-ground extinction coefficients suddenly increased; meanwhile, the PM_2.5_ concentration and SIA level also rapidly increased and reached peak values (179 μg/m^3^ for PM_2.5_). From 00:00, February 20 to 12:00, February 21, the PBLH was slightly uplifted, and wind speed increased. Then, the vertical diffusion conditions improved, and the level of pollutants consequently declined. Afterwards, the PBLH decreased, the wind speed decreased from 12:00 to 22:00 on February 21, and the PM_2.5_ and SIA levels continued to increase, reaching their peaks. From 0:00 to 21:00 on February 22, the diffusion capability was relatively good, and PM_2.5_ and SIAs decreased gradually; however, at 22:00, the near-ground distinction coefficient suddenly increased, and PM_2.5_ and SIAs rose and reached a peak value again at 0:00 on February 23. Then, PM_2.5_ and SIAs gradually decreased with atmospheric diffusion, and at 0:00 on February 23, the extinction coefficient rose again, while PM_2.5_ and SIAs climbed slowly afterwards. Finally, in the PDP, the boundary layer was uplifted, the wind speed became stronger, and the overall atmospheric diffusion condition improved with a relatively small amount of pollutant emissions near the ground; thus, the levels of PM_2.5_ and SIA declined in a stepwise manner and were maintained at relatively low levels.

It could be concluded that near-ground PM_2.5_ accumulated as PBLH significantly decreased; and other factors, including the increase in moisture level, higher RH and local straw burning, also resulted in slight pollution. Additionally, the extinction coefficient would have increased with increasing PM_2.5_. PBLH would have further lowered, and with the increase in RH, more moisture would have been absorbed by PM_2.5_, which could have enhanced gas-particulate formation to generate more SIAs. Previous studies^53–55^ have proposed that high RH levels enhance the reaction rates of secondary aerosols. Overall, the high RH, low PBLH, and poor horizontal and vertical atmospheric diffusion capacities combined with the effects of local straw burning gradually worsened the pollution. Thus, on February 24, the PBLH was obviously uplifted, the wind speed increased, the diffusion factors improved, and the air quality improved.

### Analysis of pollutants from regional transportation

**Figure 6:**
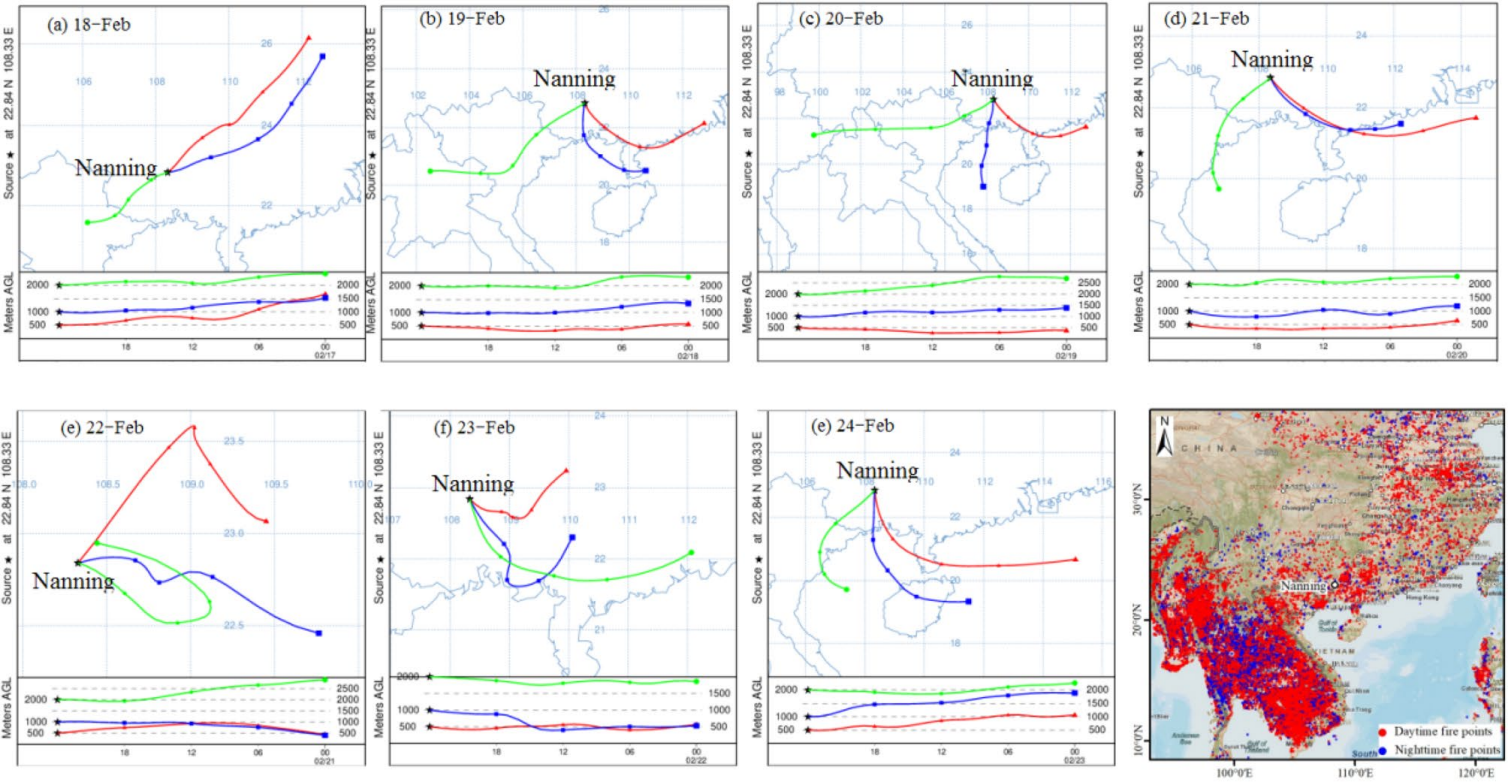
24 h backward trajectory figure from 18 to 24th February, 2020 in Nanning (Fire Spots Map of Southern China and Southeast Asia was cited from NASA data, https://firms.modaps.eosdis.nasa.gov/).

It is well known that pollutants generated by BB can be transported long distances^56,57^. As there were many fire spots identified in the regions around Nanning City, the pollution from regional transportation should also be considered. The HYSPLIT model proposed by NOAA (https://www.arl.noaa.gov/) was adopted to analyse the airflow back trajectory of pollutants during the sampling period. Airflows at heights of 500 m, 1000 m and 2000 m were selected to calculate the backward trajectory figure (Fig. 6a–d). From February 18 to 21, the air masses at 500 m and 1000 m were mainly influenced by the air flows from Guangdong Province and Beibu Gulf located in a southeastern direction, while air masses at 2000 m were mainly influenced by the airflow from Vietnam. Based on the satellite remote sensing monitoring maps (Fig. 6.), it was found that many fire spots occurred in Vietnam, Laos, Thailand and Cambodia during the period when the pollution event occurred in Nanning. The backward trajectory calculations indicated that the long-distance transportation of biomass incineration pollutants from these countries would have had some influence on the regional atmosphere in Nanning. Recently, Yue et al.^58^ showed that the CO level near the ground decreased by 17% compared with the same period in the previous year, while the concentration of CO in the troposphere increased by 2.5%. These results also supported the previous conclusion that long-distance transportation of biomass incineration from foreign countries could have affected the atmosphere in southern China. In this study, an increase in CO levels during the PAP in Nanning was identified, and it was also believed to be due to the influence of BB both in regional cities and in Southeast Asia. Interestingly, on February 22 (Fig. 6e), it was shown that the origins of air masses at 500 m, 1000 m and 1500 m were from localities within Nanning. Based on the analysis of meteorological land weather conditions, it was found that the pollutants accumulated as a wind convergence zone formed over Nanning that day. However, on February 23 (Fig. 6f), the airflow was from cities that included Laibin and Guigang, which had relatively intense fire spots; on February 24 (Fig. 6g), the airflows at the three heights were mainly from neighbouring Guangdong Province and Beibu Gulf, and as the meteorological diffusion conditions had been improving, pollution began to gradually dissipate. Overall, based on the analysis of fire spot contribution maps and backward trajectories, it was concluded that the air masses had passed through the regions with intensive fire spots, and the PM_2.5_ pollution event in Nanning was generated not only from the local BB but also from transportation from surrounding countries in Southeast Asia.

## Conclusion

In this work, we comprehensively investigated the sources and causes of PM_2.5_ pollution that occurred in Nanning, Guangxi, during the COVID-19 lockdown in February 2020. Three haze stages were categorized as the PPP, PAP and PDP, with average hourly PM_2.5_ concentrations of 32, 73 and 35 μg/m^3^, respectively. The dominant source of PM_2.5_ in the PPP was BB (40.4%), followed by secondary inorganic sources (28.1%) and motor vehicle exhaust (11.7%). The PAP was characterized by a large abundance of secondary inorganic sources, which contributed 56.1% of the total PM_2.5_ concentration, followed by BB (17.4%). The significant increase in secondary inorganic sources could have been due to the high concentrations of NO_2_ and NH_3_, which enhanced the liquid-phase oxidation of SO_2_ under relatively high humidity. Interestingly, the absorption Ångström exponent (2.2) in the PPP was higher than those in the other two periods, and based on the analysis of fire spots monitored by remote satellite sensing, aerosol absorptions in Nanning City could have been influenced by the burning of both biomass and fossil fuel. Additionally, pollutants produced by the BB accumulated and caused haze events in the PAP, which was partially due to unfavourable meteorological conditions such as increased humidity and decreased PBLH. The PBL–RH–SPM–PM positive feedback mechanism was employed to elucidate the atmospheric processes in this study, which indicated that it is an ideal model to explain the occurrence and development of pollution. Finally, this study highlights the importance of understanding the role of BB, secondary inorganic sources and meteorology to call for policies for emission control strategies.

## Material and methods

### Description of the sampling site

**Figure 7:**
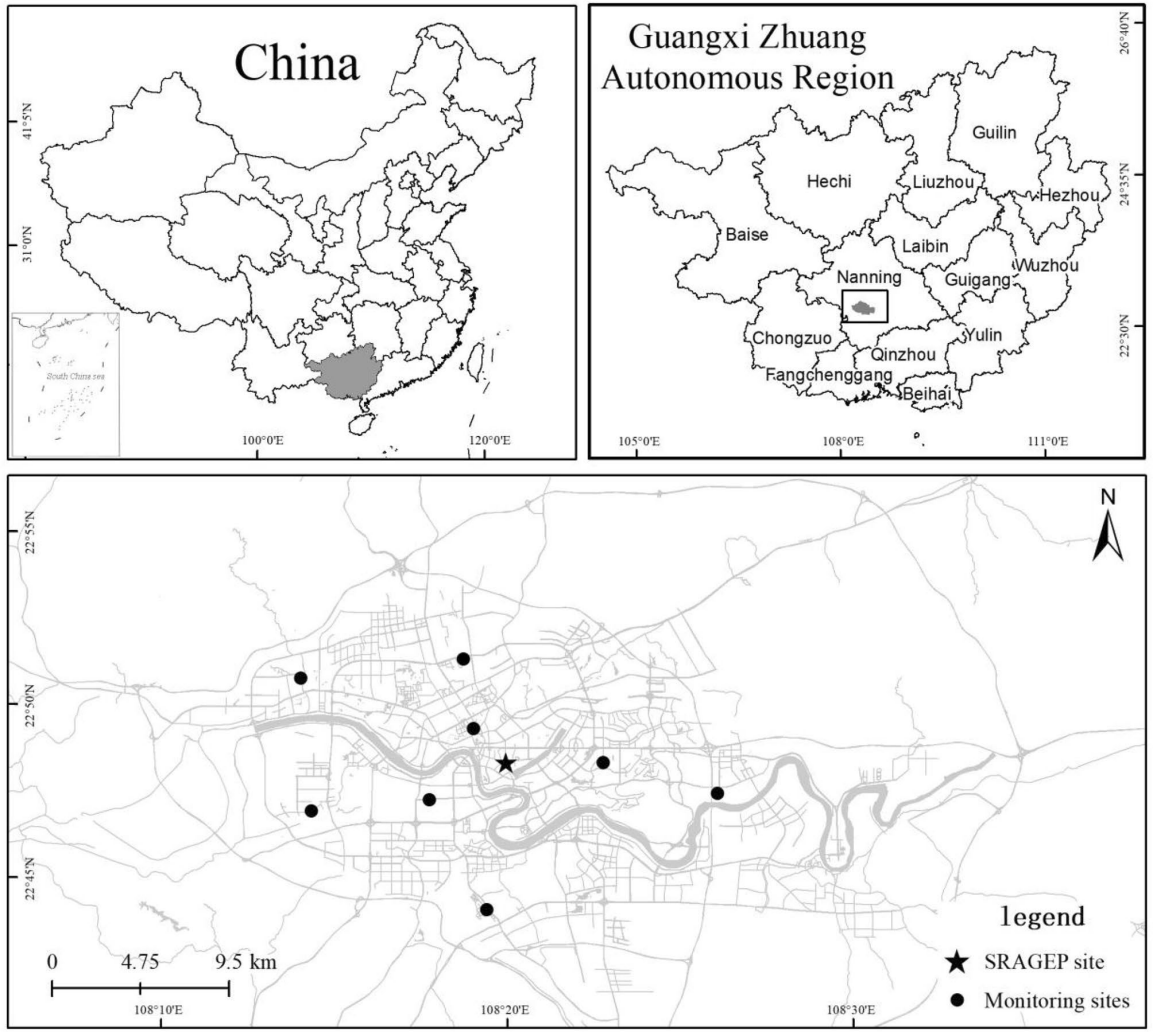
Schematic diagram for the locations of observation stations. The map was generated using ArcGIS Desktop 10.3, https://desktop.arcgis.com/en.

Located in southern China, Nanning is the provincial capital of Guangxi (Fig. 7). Ambient measurements, including single particulate aerosol mass spectrometry (SPAMS), aethalometry (AE-31), particulate Lidar and solar radiometry, were conducted at the atmospheric observation station at the Scientific Research Academy of Guangxi Environmental Protection (SRAGEP 22.8067° N, 108.3335° E), Nanning, China, which is located at a height of approximately 30 m above the ground and surrounded by heavy traffic, residential areas and restaurants. Additionally, there are eight national air-quality monitoring stations in Nanning, and Fig. 7 shows the locations of our sampling site and the eight national stations.It should be noted that the sampling site was located in the Nanning centre region; therefore, it was representative of the Nanning urban area.

### Measurements of pollutants

PM_2.5_ was measured using the combined methods of aerosol light scattering and beta ray attenuation (Synchronized Hybrid Ambient Real-time Particulate Monitor, 5030i, Thermo Scientific), and PM_10_ measurements were performed by using the beta attenuation method (Continuous Particulate Monitor, FH 62 C14, Thermo Scientific). The concentrations of CO, NO, NO_2_, and SO_2_ were monitored by infrared radiation absorption (CO Analyser, 48i, Thermo Fisher Scientific), chemiluminescence (NO–NO_2_–NO*x* Analyser, 42i-HL, Thermo Scientific) and pulsed UV fluorescence methods (SO_2_ analyser, 43i, Thermo Scientific), respectively. All of the above data were recorded in real time 24 h a day with continuous sampling.

### Single particulate aerosol mass spectrometer (SPAMS)

Single particulate aerosol mass spectrometry (SPAMS, Hexin Analytical Instrument Co., Ltd., China) has been employed to monitor both the diameter and chemical compositions of single particulate aerosols^59,60^, and the applications of the SPAMS have been widely described in many published studies^61–64^. Briefly, ambient particles were introduced into the SPAMS with a sampling flow rate of 75 mL/min. Aerodynamic lenses were used to achieve the focus of accelerated single aerosol particulates, and the aerodynamic diameters of particles 0.13–3 μm in size could be measured by scattering signals of two continuous Nd:YAG laser beams. A laser (wavelength of 266 nm with an energy of 0.5–0.6 mJ and energy density of 108 W/cm) was simultaneously triggered to ionize the measured particle, and the generated cations and anions were analysed by dual polarity time-of-flight mass spectra. Generally, the range of particle diameters could be analysed at 0–2.5 μm with a rate of 20 particles/s and a bombarded rate of > 20%. The MS resolution was better than 500 full width at half maximum (FWHM), and the measurable range of chemical composition (molecular weight) was 1–500 u.

According to the characteristics of pollutants from different sources, their characteristic ion peaks, and the Technical Guide for Source Analysis of Atmospheric Particulate Matter^65^, combined with the local energy consumption structure, environmental pollutant characteristics, the tracer ion method was adopted to divide the sources of particulates into eight categories, including cooking, dust, biomass burning (BB), vehicle exhaust, coal, industrial processes, secondary inorganic sources and others. The tracer ion method is based on a specific component or source containing characteristic ion peaks, and the particulate matter is classified according to these characteristic ion peaks. In this study, the coal sources included particles discharged from bulk coal, boilers and power plants. The cooking source refers to particles containing oleic acid, which comes from catering lamp black. Dust particles have sources that include buildings, roads, and crustal dust particles, mainly containing mineral compositions. BB particles were produced by the open air incineration of biofuels and straw, which are mainly characterized by levoglucosan fragment ions. Vehicle exhaust contains emission particles from diesel and gasoline vehicles. Coal sources include particles discharged from coal boilers and coal-fired power plants. Industrial process sources include particles discharged from chemical, cement, and metal smelting processes. Secondary inorganic sources mainly refer to particles containing only secondary inorganic ions, such as nitrate, sulphate and ammonia, except for potassium ions. These particles can reflect the secondary reaction intensity of atmospheric particles to a certain extent. Particles that were not included in the source class above and whose origin could not be identified were classified as other sources. The main characteristic pollutant components of particulate matter and their ion peaks are shown in Table S3.

### Aethalometer

Particles collected on quartz fibre filters (QFFs) were analysed by an aethalometer (AE-31, Magee Scientific, Inc., USA) at seven measurement wavelengths (370, 470, 520, 590, 660, 880, and 950 nm). The attenuation (*ATN*) of the light beam transmitted through the sample was measured, and the b_ATN_ of the particulate time zone was calculated based on the change rate of *ATN* (Δ*ATN*) by the equations below^66^:

1 $$ ATN = \ln \left( {\frac{{I_{o} }}{I}} \right) $$

2 $$ {\text{b}}_{ATN} = \frac{\Delta ATN \times A}{{100 \times \Delta t \times Q}} $$

where I_0_ and I are the light intensities before and after the light beam passes through the filter membrane (W/cm^2^), respectively. A is the area of the spot on the quartz belt, cm^2^. Δt is the reading period (5 min used in this study). *Q* is the air flow rate, L/min (4.9 L/min in this study). When particles accumulated in one spot on the filter belt reached the largest attenuation value of − 125 (λ at 370 nm), the belt was shifted to the next spot, and the analysis was initialized; thus, the testing processes were automatically completed in cycled periods. The light absorption coefficient of aerosols (σ_abs_) was calculated based on the measured light attenuation at seven wavelengths, as shown in the published literature^40,67,68^. Determination of the absorption Ångström exponent (AAE) was calculated based on previous studies^40,69^.

### Particulate Lidar

The application of particulate Lidar is based on the return signal due to elastical backscatter by atmospheric particles, and the processes can be expressed by Eq. (3) as follows:

3 $$ P(R) = \frac{{E_{O} \eta L}}{{R^{2} }}O(R)\beta $$

where *P (R)* is the energy received by the Lidar from the backscattering signals of the atmosphere at altitude R; Eo is the emission energy of the laser; ηL is the overall efficiency of the optical and detected parts of the Lidar system; *O (R)* is the laser overlap factor; and *β (R)* and *α (R)* represent the backscatter coefficient and extinction coefficient of the atmosphere, respectively. Generally, different approximation methods have been proposed to calculate the actual *β (R)* and *α (R)* in the literature, and the “Fernald method” has been widely adopted among all of them^70,71^. The Lidar system (AGHJ-I-LIDAR, Wuxi CAS Photonics Co., Ltd.) used in this study was composed of a laser emission unit, optical receiving unit and signal acquisition unit. The laser emission unit is mainly composed of laser and emission telescopes. The detection wavelengths were 355 nm and 532 nm, and the single pulse energy was approximately 20 mJ. The optical receiving system consisted of a Cassegrain telescope, a narrow-band filter and a photodetector. The laser emitting unit emitted 355 nm and 532 nm detective pulses to the target area, and the pulses were scattered by atmospheric aerosols, particles or clouds in the transmission path. Then, the backscattered light was received by the receiving telescope, and the backscattered signal light successively passed through a small aperture, collimator and spectroscope and divided into two signals with wavelengths of 355 nm and 532 nm, which were received and finally converted to the corresponding electrical signals by a photomultiplier after polarizing the prism.

### Planetary boundary layer heights and meteorological factors

Planetary boundary layer heights were observed by a Lidar system (AGHJ-I-LIDAR, Wuxi CAS Photonics Co., Ltd.). Meteorological factors, including precipitation, temperature (T), relative humidity (RH), air pressure (P), wind speed (WS), wind direction (WD), visibility (V) and total solar radiation, were monitored by a solar radiometer (Beijing Huatron Technology Development Co., Ltd.) with a temporal resolution of 1 min. The hourly average of each factor was adopted in this study.

## Supplementary Information

Supplementary Information 1.
