# Supplementary material for: Cause analysis of PM2.5 pollution during the COVID-19 lockdown in Nanning, China
Source: Sci Rep. 2021 May 27;11:11119. doi: 10.1038/s41598-021-90617-5 (PMC8160135; doi:10.1038/s41598-021-90617-5)
Supplement: Supplementary file 1 — Supplementary Information 1. [file 41598_2021_90617_MOESM1_ESM.docx]

[**Supplementary**](https://www.ncbi.nlm.nih.gov/pmc/articles/PMC7235082/bin/41598_2020_65187_MOESM1_ESM.docx) **Materials**

**Cause Analysis of PM_2.5_ pollution** **during the COVID-19 lockdown in Nanning, China**

Zhaoyu Mo ^1,2 a^, Jiongli Huang ^2,3a^, Zhiming Chen ^2^, Bin Zhou ^1*^, Kaixian Zhu ^2^, Huilin Liu ^2^, Yijun Mu ^2^, Dabiao Zhang ^2^, Shanshan Wang ^1^

^1^ Shanghai Key Laboratory of Atmospheric Particle Pollution and Prevention, Department of Environmental Science and Engineering, Fudan University, Shanghai 200433, China

^2^ Atmospheric Environment Research Center, Scientific Research Academy of Guangxi Environmental Protection, Nanning 530021, China

^3^ Department of Occupational Health and Environmental Health, School of Public Health, Guangxi Medical University, Nanning 530021, China

* Corresponding author: Bin Zhou, E-mail: binzhou@fudan.edu.cn, Address: Shanghai Key Laboratory of Atmospheric Particle Pollution and Prevention, Department of Environmental Science and Engineering, Fudan University, No. 220 Handan Road, Shanghai 200433, China.

^a^ These authors have contributed equally to this article.


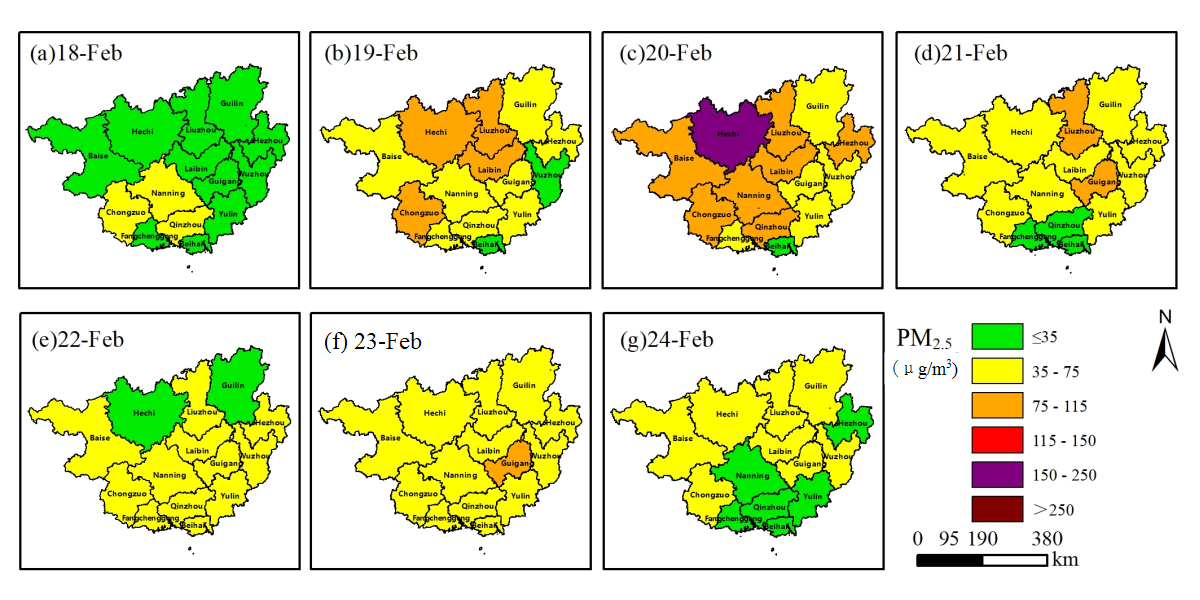


**Fig. S1** Daily average PM_2.5_ distributions of cities in Guangxi, February18-24, 2020.

The map was generated using ArcGIS Desktop 10.3, https://desktop.arcgis.com/en. (Data source: http:// 106. 37. 208. 233: 20035)


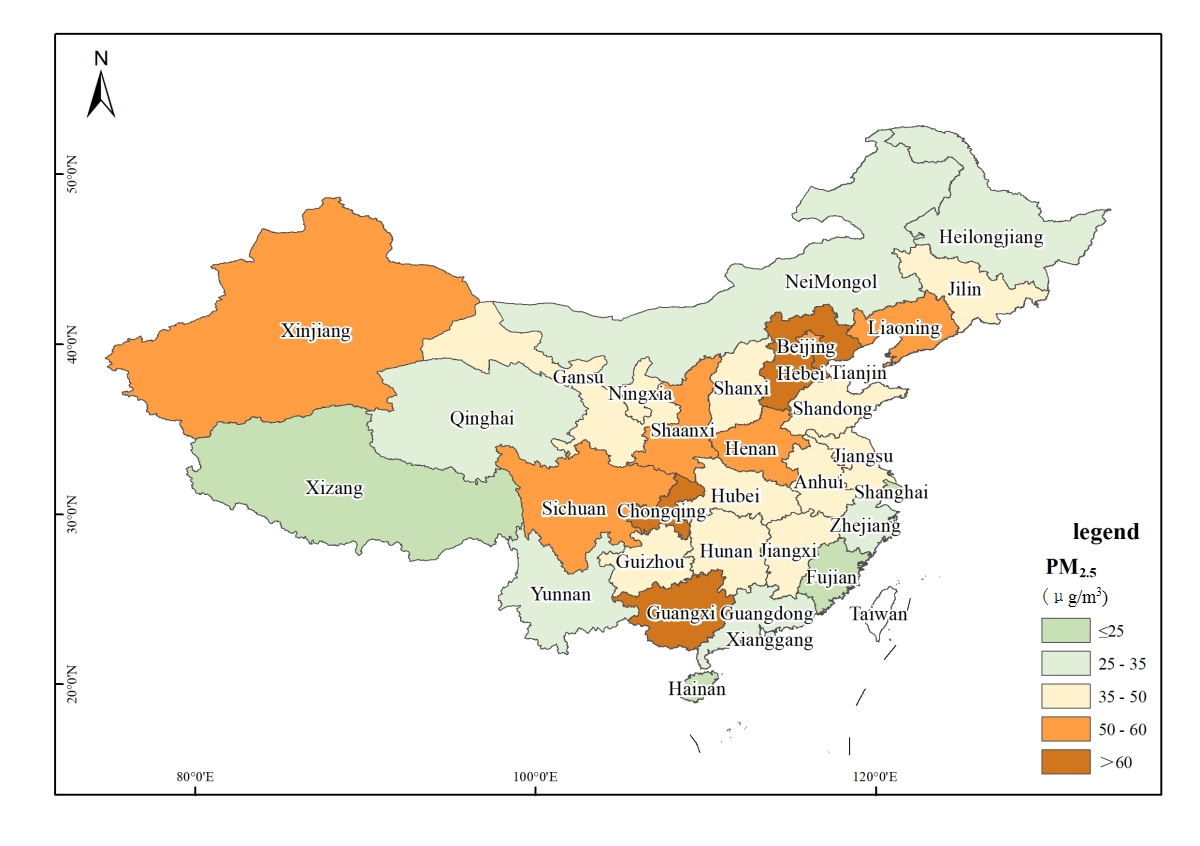


**Fig. S2** The average PM_2.5_ concentrations of all provinces of China, 19-23, Feb, 2020.

The map was generated using ArcGIS Desktop 10.3, https://desktop.arcgis.com/en. (Data source: http:// 106. 37. 208. 233: 20035)

**Table S1** Comparison of ambient air quality data of Nanning City released by China Environmental Monitoring General Station (CNEMC) with the observation data in our SRAGEP station.

| Date | AQI | |  | PM_2.5_  (μg/m^3^) | |  | PM_10_  (μg/m^3^) | |  | SO_2_  (μg/m^3^) | |  | NO_2_  (μg/m^3^) | |  | O_3_  (μg/m^3^) | |  | CO  (mg/m^3^) | |  |
| --- | --- | --- | --- | --- | --- | --- | --- | --- | --- | --- | --- | --- | --- | --- | --- | --- | --- | --- | --- | --- | --- |
|  | CNEMC | SRAGEP |  | CNEMC | SRAGEP |  | CNEMC | SRAGEP |  | CNEMC | SRAGEP |  | CNEMC | SRAGEP |  | CNEMC | SRAGEP |  | CNEMC | SRAGEP | |
| 18-Feb | 52 | 70 |  | 36 | 33 |  | 41 | 40 |  | 10 | 15 |  | 14 | 12 |  | 101 | 128 |  | 0.5 | 0.597 | |
| 19-Feb | 84 | 83 |  | 62 | 61 |  | 71 | 64 |  | 9 | 15 |  | 20 | 17 |  | 76 | 97 |  | 0.7 | 0.838 | |
| 20-Feb | 120 | 133 |  | 91 | 101 |  | 106 | 111 |  | 9 | 14 |  | 26 | 22 |  | 86 | 124 |  | 0.9 | 1.09 | |
| 21-Feb | 62 | 70 |  | 44 | 49 |  | 59 | 57 |  | 7 | 13 |  | 19 | 15 |  | 107 | 136 |  | 0.7 | 0.874 | |
| 22-Feb | 85 | 98 |  | 63 | 73 |  | 79 | 81 |  | 7 | 13 |  | 23 | 20 |  | 121 | 160 |  | 0.8 | 1.085 | |
| 23-Feb | 80 | 93 |  | 59 | 62 |  | 76 | 72 |  | 8 | 13 |  | 25 | 21 |  | 134 | 157 |  | 0.8 | 1.07 | |
| 24-Feb | 50 | 61 |  | 31 | 33 |  | 50 | 49 |  | 8 | 13 |  | 15 | 11 |  | 98 | 128 |  | 0.7 | 0.886 | |

**Fig. S3** Correlation between (a) PM_2.5_ concentration released by CNEMC and PM_2.5_ concentration observed at the SRAGEP site; (b) CO concentration released by CNEMC and COconcentration observed at the SRAGEP site. (Data source of CNEMC: http:// 106. 37. 208. 233: 20035)

Table S2. Pearson correlation coefficient of sulfate particulates, SO_2_, NO_2_, NH_3_ and RH during different stages in the study.

| Stages |  | SP | SO_2_ | NO_2_ | NH_3_ | RH |
| --- | --- | --- | --- | --- | --- | --- |
| Total | SP | 1 |  |  |  |  |
|  | SO_2_ | 0.029 | 1 |  |  |  |
|  | NO_2_ | 0.537** | 0.077 | 1 |  |  |
|  | NH_3_ | -0.007 | 0.413** | 0.014 | 1 |  |
|  | RH (%) | 0.516** | -0.375** | 0.330** | -0.181* | 1 |
| NPAP | SP | 1 |  |  |  |  |
|  | SO_2_ | -0.327* | 1 |  |  |  |
|  | NO_2_ | -0.002 | 0.094 | 1 |  |  |
|  | NH_3_ | -0.480** | 0.636** | -0.243 | 1 |  |
|  | RH | 0.346** | -0.470** | -0.015 | -0.864** | 1 |
| PAP | SP | 1 |  |  |  |  |
|  | SO_2_ | 0.240* | 1 |  |  |  |
|  | NO_2_ | 0.454** | 0.147 | 1 |  |  |
|  | NH_3_ | 0.116 | 0.317** | 0.098 | 1 |  |
|  | RH | 0.474** | -0.274** | 0.350** | 0.243* | 1 |

**Notes:**

SP = Sulfate particulates.

^*^ Correlation is significant at the 0.05 level (2-tailed).

^**^ Correlation is significant at the 0.01 level (2-tailed).

Table S3. The main characteristic pollutant components of particulate matter and their ion peaks.

| Source type | Ion peaks and characteristic components | Source analysis |
| --- | --- | --- |
| Dust ^1-3^ | 27 [Al^+^], 40 [Ca^+^], 56 [CaO^+^ or Fe^+^], -60 [SiO- 2], -76 [SiO- 3], -16 [O^-^], -17 [OH^-^], 48[Ti^+^], 64 [TiO^+^], 96 [Ca_2_O^+^], -79 [PO- 3] | Buildings, roads, and crustal dust particle |
| BB ^4-6^ | 39 [K^+^], 12 [C^+^], 36 [C+ 3], 37 [C_3_H^+^], -26 [CN^-^], 113/115 [K_2_Cl^+^], 97 [K_3_SO+ 3], 213 [K_3_SO+ 4], 27 [C_2_H+ 3], levoglucosan [m / z = -45, -59, -71], -42 [CNO^-^] | Open air incineration of biofuels and straw |
| Vehicle exhaust ^7-8^ | 40 [Ca^+^], -79 [PO- 3], -62 [NO- 3], Cn^±^ [n > 0] | Diesel vehicles and gasoline vehicles |
| Coal ^9-10^ | 27 [C_2_H+ 3], 7 [Li^+^], 39 [K^+^], -32 [S^-^], -64 [SO- 2], -80 [SO- 3], high molecular mass [m /z = 178, 189, 202, 228, 252, 276], Cn^±^ [n = 1, 3, 4, 5], OC [m /z = -43, -63, -79] | Coal boilers and coal-fired power plants |
| Industrial process ^11-12^ | 63/65 [Cu^+^], 64 / 66 / 68 [Zn^+^], 99 / 101 / 103 [ZnCl^+^], 206 / 207 / 208 [Pb^+^], -35 [Cl^-^], EC, OC, -76 [SiO- 3], -79 [PO- 3] | Coal boilers and coal-fired power plants |
| Secondary inorganic source ^13-14^ | -97 [H_2_SO- 4], -62 [NO- 3], -46 [NO- 2], 18 [NH+ 4] | Secondary precursor transformation |
| Cooking | m/z= -115/-117/-131/-145/-159/-173/-187/-121/-281/-255/-283/-147 | Catering lampblack |

**References**

1. Silva, P. J., Carlin, R. A. & Prather, K. A. Single particle analysis of suspended soil dust from Southern California. *Atmos. Environ.* **34**, 1811-1820 (2000). https://doi.org/10.1016/S1352-2310(99)00338-6

2. Sullivan, R. C., Guazzotti, S. A., Sodeman, D. A. & Prather, K. A. Direct observations of the atmospheric processing of Asian mineral dust. *Atmos. Chem. Phys.* **7**, 1213-1236 (2007). https://doi.org/10.5194/acp-7-1213- 2007

3. Sullivan, R. C. *et al*. Mineral dust is a sink for chlorine in the marine boundary layer. *Atmos. Environ.* **41**, 7166-7179 (2007). https://doi.org/10.1016/j.atmosenv.2007.05.047

4. Hudson, P. K. *et al*. Biomass - burning particle measurements: Characteristic composition and chemical processing．*J. Geophys. Res.: Atmos.* **109**, D23S27 (2004). https://doi.org/10.1029/2003JD004398

5. Silva, P. J., Liu, D. Y., Noble, C. A. & Prather, K. A. Size and chemical characterization of individual particles resulting from biomass burning of local Southern California species．*Environ. Sci. Technol.* **33**, 3068-3076 (1999). https://doi.org/10.1021/es980544p

6. Yang, F. *et al*. Single particle mass spectrometry of oxalic acid in ambient aerosols in Shanghai: Mixing state and formation mechanism. *Atmos. Environ.* **43**, 3876-3882 (2009). https://doi.org/10.1016/j.atmosenv.2009.05.002

7. Moffet, R. C. & Prather, K. A. In-situ measurements of the mixing state and optical properties of soot with implications for radiative forcing estimates. *Proc. Natl. Acad. Sci. U. S. A.* **106**, 11872-11877 (2009). https:// doi.org/10.1073/pnas.0900040106

8. Spencer, M. T. & Prather, K. A. Using ATOFMS to determine OC/EC mass fractions in particle. *Aerosol Sci. Technol.* **40**, 585-594 (2006). https://doi.org/10.1080/02786820600729138

9. Guazzotti, S. A. *et al*. Characterization of carbonaceous aerosols outflow from India and Arabia: Biomass / biofuel burning and fossil fuel combustion. *J. Geophys. Res.: Atmos.* **108**, 4485 (2003). https: //doi.org/10.1029/ 2002JD003277

10. Spencer, M. T., Holecek, J. C., Corrigan, C. E., Ramanathan, V. & Prather, K. A. Size-resolved chemical composition of aerosol particles during a monsoonal transition period over the Indian Ocean. *J. Geophys. Res.: Atmos.* **113**, D16305 (2008). https://doi.org/10.1029/2007JD008657

11. Moffet, R. C., de Foy, B., Molina, L. T., Molina, M. J. & Prather, K. A. Measurement of ambient aerosols in northern Mexico City by single particle mass spectrometry. *Atmos. Chem. Phys.* **8**, 4499-4516 (2008). https:// doi.org/10.5194/acp-8-4499-2008

12. Moffet, R. C. *et al*. Characterization of aerosols containing Zn, Pb, and Cl from an industrial region of Mexico City. *Environ. Sci. Technol.* **42**, 7091-7097 (2008). https://doi.org/10.1021/es7030483

13. Huang, K. *et al*. Typical types and formation mechanisms of haze in an Eastern Asia megacity, Shanghai. *Atmos. Chem. Phys.* **12**, 105-124 (2012). https://doi.org/10.5194/acp-12-105-2012

14. Fairlie, T. D. *et al*. Impact of mineral dust on nitrate, sulfate, and ozone in transpacific Asian pollution plumes. *Atmos. Chem. Phys.* **10**, 3999-4012 (2010). https://doi.org/10.5194/acp-10-3999-2010
